# Role of streptococcal infection in the etiopathogenesis of pityriasis lichenoides chronica and the therapeutic efficacy of azithromycin: a randomized controlled trial

**DOI:** 10.1007/s00403-022-02398-0

**Published:** 2022-09-21

**Authors:** Amira Elbendary, Randa Youssef, Mona R. E. Abdel-Halim, Dalia Abdel Halim, Dina Ahmed El Sharkawy, Mostafa Alfishawy, Maha A. Gad, Abdallah Gad, Maha Fathy Elmasry

**Affiliations:** 1 Dermatology Department, Kasr Alainy Faculty of Medicine, Cairo University, Kasr Al Ainy St, Cairo, 11562 Egypt; 2 Infectious Diseases Consultants and Academic Researchers of Egypt (IDCARE), Cairo, Egypt; 3 Clinical Pathology Department, Kasr Alainy Faculty of Medicine, Cairo University, Cairo, Egypt; 4 Lowell General Hospital, Lowell, MA USA; 5 Biostatistics and Cancer Epidemiology Department, National Cancer Institute, Cairo University, Cairo, Egypt

**Keywords:** Randomized controlled trial, Pityriasis lichenoides chronica, Azithromycin, NB-UVB, Streptococcal infection, ASOT, Hypopigmented

## Abstract

The exact aetiology of pityriasis lichenoides chronica (PLC) remains unknown. While phototherapy is the most investigated therapeutic modality, azithromycin has been used scarcely. The aim of this study is to evaluate the therapeutic efficacy of azithromycin in the treatment of PLC compared to NB-UVB and evaluating the presence of streptococcal infection as a possible etiological factor in PLC patients. The study was designed as a randomised controlled trial. Twenty-four patients with PLC were randomly allocated into either azithromycin (*n* = 13, standard dose every 10 days) or NB-UVB (*n* = 11, thrice weekly) groups. End of study (EOS) was either complete clearance of lesions or a maximum of 8 weeks. Therapeutic efficacy was defined as percent reduction in lesions and was calculated for the rash as a whole, erythematous papules alone, and hypopigmented lesions alone and graded into complete, very-good, good, poor or no response. Anti-streptolysin O titre (ASOT), anti-deoxyribonuclease B titre (anti-DNaseB) and throat culture were evaluated at day 0. No significant difference existed between both groups as regards therapeutic efficacy. At EOS, NB-UVB achieved significantly more percent reduction in the extent of hypopigmented lesions and consequently in the rash as a whole (*p* = 0.001, *p* = 0.034, respectively). The extent of the rash as a whole was significantly less in the NB-UVB at EOS (*p* = 0.029, respectively). The effect of NB-UVB on hypopigmented lesions appeared early at week 4 of treatment. Only two patients, one from each group, relapsed during the 3 month follow-up. Evidence of recent streptococcal infection was present in 79% of the cases, mainly in the form of elevated ASOT (94.7%). It was significantly more encountered in young children (< 13 years) (*p* = 0.03) and was associated with more extent of erythematous papules and consequently with more extent of the rash as a whole (*p* = 0.05 and *p* = 0.01, respectively). It did not affect outcome of therapy at EOS. Azithromycin did not show more favorable response in patients with recent streptococcal infection. Therapeutic efficacy of azithromycin is comparable to NB-UVB in treatment of PLC; however, NB-UVB is superior in management of hypopigmented lesions. It is highly suggested that PLC could be a post streptococcal immune mediated disorder.

*Registration number*: ClinicalTrials.gov, NCT03831269.

## Introduction

Pityriasis lichenoides chronica (PLC) is the chronic form in the spectrum of pityriasis lichenoides [1]. The predominant clinical feature of PLC is a reddish-brown papule with mica-like scale. Hypopigmented macules are also present, and they may be predominant specially in dark skinned individuals [2–4].

The etiopathogenesis of PLC remains uncertain with three major theories being proposed: a lymphoproliferative, an infectious; and an immune complex mediated theory [5]. Characteristics of pityriasis lichenoides that are consistent with an infectious model include the acute eruptive nature, young age at onset, and reports of familial outbreaks and disease clustering in communities [6]. Elevated titers of certain pathogens and clearing of the disease after pathogen-specific treatment have been reported further substantiating this theory [7].

To date, there is no standard treatment of PLC. Several lines of treatments have been tried with variable results including topical agents, antibiotics and immunosuppressives. Combination therapy is considered the best approach by some authors [8].

The aim of this study was to assess the therapeutic efficacy of azithromycin in comparison with NB-UVB in PLC patients and investigating the presence of streptococcal infection in PLC patients and its therapeutic implications.

## Patients and methods

### Study design

This prospective randomized controlled trial (RCT) study was conducted at the Dermatology Department Outpatient Clinic, Faculty of Medicine, Cairo University (Kasr Al Aini hospital) during the period from February 2019 to July 2020. The study design was approved by both the Department Scientific and Ethical Committee Boards and informed consents were signed by all participants or guardians for participation in the study. This study was registered in www.clinicaltrials.gov under the number NCT03831269.

### Participation

Given the rarity of the disease, a 12-month recruitment period was assigned. During this period, 30 patients with PLC (confirmed histopathologically) presenting consecutively to the Dermatology Outpatient Clinic were assessed based on certain inclusion and exclusion criteria for their eligibility to be enrolled in the study.

Assessment included: complete medical history [personal history, history of present illness, history of relevant allergies (specifically to azithromycin), photosensitivity, chronic medical illnesses, heart diseases and drug history (specifically photosensitizing drugs)] and routine laboratory work up [complete blood count (CBC), liver function tests (aspartate transaminase, AST and alanine transaminase, ALT) and kidney function tests (urea and creatinine)].

Included in the study were patients from either sex with age of 6 years or more who presented with classic papular eruption of PLC (with or without associated hypopigmented lesions). Excluded from the study were patients presenting with only hypopigmented macules (with PLC-like picture in their skin biopsy), patients with PLC associated with mycosis fungoides (MF), patients with known absolute contraindications to NB-UVB, patients with impaired liver or kidney functions, patients with history of any heart disease as well as patients with known hypersensitivity to azithromycin.

Accordingly, 24 patients were enrolled in the therapeutic phase of the trial. A wash out period of at least 1 month from any topical or systemic treatment that can affect the course of PLC was adopted (including steroids, antibiotics, or phototherapy).

### Intervention

Patients were randomly allocated into either one of the arms of the trial. A 1:1 assignment was adopted; however, one patient who was reallocated from the NB-UVB arm to the azithromycin arm due to personal circumstances that would affect his compliance. Accordingly, the azithromycin arm contained 13 patients and the NB-UVB arm contained 11 patients.

The dose of azithromycin was 10 mg/kg/day oral suspension for 3 consecutive days for pediatric patients (< 12 years) and 500 mg/day for 3 consecutive days for adults. The cycle was repeated every 10 days for 3 cycles (3 cycles/month) and was repeated when required according to patient’s response up to 6 cycles (Youssef and Elmasry, unpublished data).

Patients allocated for NB-UVB phototherapy received sessions according to the protocol of the Phototherapy Unit, Dermatology Department, Cairo University, where an initial dose was dependent on the minimal erythema. The dosage was then increased according to the patient’s response. The sessions were given 3 times weekly.

No concomitant therapy was allowed except for Vaseline topical daily application (if needed).

### End of study (EOS)

End of study (EOS) was either achieving complete clearance of lesions or a maximum of 8 weeks for both arms (6 cycles of azithromycin and 24 sessions for NB-UVB).

### Assessments of enrolled patients

Detailed skin examination was carried out by blinded investigators (DA and MFE). Anatomical sites affected were documented. The extent of erythematous papular lesions, hypopigmented lesions and the rash as a whole was calculated at day 0, week 2, week 4, week 6 and at EOS using the rule of hand surface area (1% of body surface area). During the biweekly follow-up, any side effects were monitored and managed accordingly.

Streptococcal infection was evaluated at day 0 by measuring anti-streptolysin O titre (ASOT) and anti-deoxyribonuclease B titre (anti-DNase B) and performing throat swab for culture and sensitivity. Streptococcal throat infection was defined by either positive ASOT, anti-DNase B titre, or positive throat culture for group A β-hemolytic streptococci [9]. Cut off values in this study were > 200 IU/ml for ASOT and > 85 IU/ml in adults and > 170 IU/ml in children for the anti-DNase titre.

### Therapeutic efficacy

Therapeutic efficacy was calculated for the erythematous papules alone, hypopigmented lesions alone and for the rash as a whole. It was defined as the percentage of reduction in the extent of the lesions and was graded as follows:

- Complete response: > 90% reduction in the extent of lesions.
- Very-good response: 75–90% reduction in the extent of lesions.
- Good response: 50–75% reduction in the extent of lesions.
- Poor response: < 50% reduction in the extent of lesions.
- No response: no reduction in the extent of lesions.
- Relapse: flaring up of an eruption after the primary skin lesions had improved during the period of the study.

### Follow-up period after EOS

After EOS, patients were followed up for 3 months.

### Statistical methods

Data analysis was performed using IBM SPSS Statistics for Windows, version 23 (IBM Corp., Armonk, N.Y., USA) (IBM, 2015). During calculation of therapeutic efficacy, intention to treat analysis was used when dealing with missed data from dropped out cases (recorded as no response) [10]. All tests were two-tailed and *p* values of less than 0.05 were considered statistically significant.

## Results

**Fig. 1:**
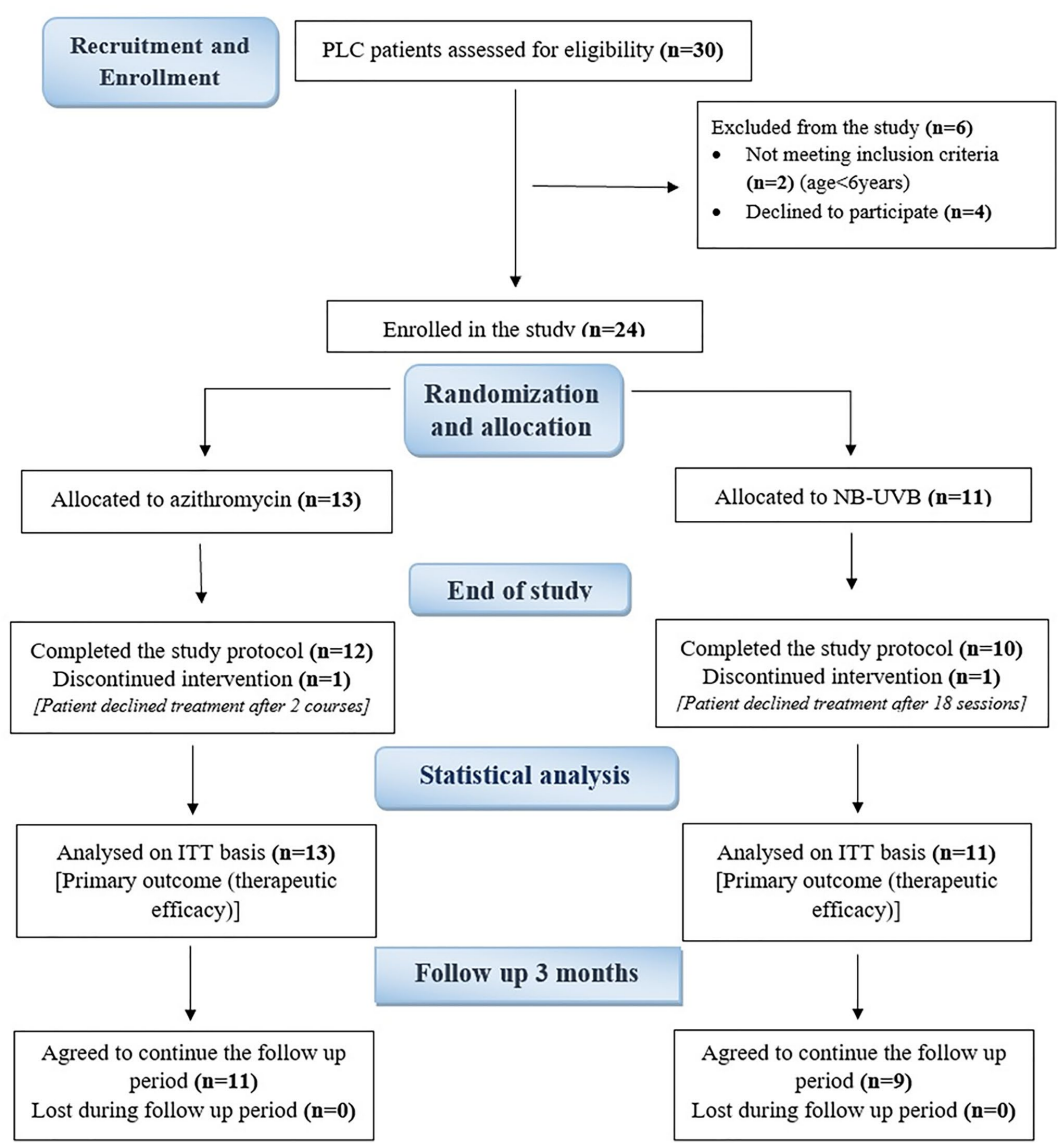
Patient flow chart demonstrating the sequence of the study according to CONSORT guidelines for reporting randomized controlled trials

Patients' flow chart is presented in Fig. 1. The study included 24 PLC patients, 13 males (54.2%) and 11 females (45.8%) with no significant gender predilection (*p* = 0.5). Their ages ranged from 6 to 68 years (median = 11, 15.08 ± 13.55). The duration of the disease ranged from 1 to 84 months (median = 21, 26.42, ± 21.03). Lesions showed central distribution pattern in 2 patients (8.3%), peripheral pattern in 3 patients (12.5%) patients, and in 19 patients (79.2%) the lesions showed diffuse pattern of distribution. Lesions involved the face in 15 patients (62.5%).

Erythematous papules were found in all 24 patients (100%), whereas hypopigmented lesions were present in 18 cases (75%) cases. The extent of the erythematous papules ranged from 0.5% to 53% (9.13 ± 10.38), whereas the hypopigmented lesions ranged from 0 to 31% (6.55 ± 7.27) and the extent of the rash as a whole ranged from 3 to 53% (15.68 ± 11.12). At baseline, both groups were statistically homogenous **(**Table 1).

**Table 1:** Comparison between both groups at baseline

	Azithromycin group (*n* = 13) (mean ± SD)	NB-UVB (*n* = 11) (mean ± SD)	*p* value
Age (years)	12 ± 5.29	18.73 ± 19.02	0.279
Sex	Females 8 (72.7%) Males 5 (38.5%)	Females 3 (27.3%) Males 8 (61.5%)	0.102
Duration (months)	27.46 ± 22.99	25.18 ± 19.49	0.795
Extent of the rash as a whole (% body surface)	18.56 ± 13.94	12.27 ± 5.27	0.152
Extent of the erythematous papules (% body surface)			
Face	0.31 ± 0.59	0.16 ± 0.32	0.443
Trunk (front)	2.13 ± 2.41	1.23 ± 1.21	0.248
Trunk (back)	1.85 ± 2.47	1.27 ± 1.12	0.463
Upper extremities	3.13 ± 4.80	1.93 ± 1.78	0.352
Lower extremities	3.75 ± 4.33	2.05 ± 1.27	0.198
Total extension	11.9 ± 13.54	6.68 ± 3.96	0.271
Extent of the hypopigmented lesions (% body surface)			
Face	0.35 ± 0.42	0.27 ± 0.39	0.662
Trunk (front)	1.63 ± 2.67	1.18 ± 1.03	0.581
Trunk (back)	1.48 ± 2.62	1.05 ± 1.01	0.589
Upper extremities	1.75 ± 2.05	1 ± 0.74	0.238
Lower extremities	2 ± 2.04	2.09 ± 1.87	0.91
Total extension	7.37 ± 9.33	5.59 ± 3.91	0.541

*p value < 0.05 is significant

### Results of the RCT

#### Evaluation of primary outcome (therapeutic efficacy)

Although no significant difference existed between both groups as regards therapeutic efficacy, the percent reduction in the extent of the rash as a whole and the extent of the hypopigmented lesions were significantly more in the NB-UVB group compared to the azithromycin group (*p* = 0.034 and *p* = 0.001, respectively). At EOS, the extent of the rash as a whole was significantly less in the NB-UVB group compared to the azithromycin group (*p* = 0.029) (Table 2).

**Table 2:** Results of the RCT

Therapeutic efficacy at EOS		Azithromycin (*n* = 13) *n* (%)	NB-UVB (*n* = 11) *n* (%)	*p* value
Rash as a whole	*Complete response*	3 (23%)	9 (81.8%)	0.83
	*Very-good response*	5 (38.6%)	1 (9%)	
	*Good response*	3 (23%)	0	
	*Poor response*	2 (15.4%)	1 (9%)	

Therapeutic efficacy at EOS		Azithromyci*n* (*n* = 13) *n* (%)	NB-UVB (*n* = 11) *n* (%)	*p* value
Erythematous Papules	*Complete response*	8 (61.5%)	9 (81.8%)	0.351
	*Very-good response*	4 (30.8%)	0	
	*Good response*	0	1 (9.1%)	
	*Poor response*	1 (7.7%)	1 (9.1%)	

Therapeutic efficacy at EOS		Azithromycin (*n* = 9) *n* (%)	NB-UVB (*n* = 9) *n* (%)	*p* value
Hypopigmented Lesions	*Complete response*	1 (10%)	8 (88.9)	0.5
	*Very-good response*	2 (20%)	0	
	*Good response*	3 (30%)	0	
	*Poor response*	3 (30%)	1 (11.1%)	

Percent reduction in the extent of rash and extent of rash at EOS	Azithromyci*n* (*n* = 12)	NB-UVB (*n* = 10)	*p* value
Percent reduction in the extent of the rash as a whole (mean ± SD)	78.13 ± 14.68	91.27 ± 13.67	0.034*
Extent of the rash as a whole at EOS (% body surface) (mean ± SD)	4.78 ± 4.89	1.2 ± 1.9	0.029*
Percent reduction in the extent of erythematous papules (mean ± SD)	90.11 ± 10.31	88.15 ± 18.24	0.785
Extent of erythematous papules at EOS (% body surface) (mean ± SD)	1.73 ± 3.31	0.66 ± 1.23	0.296
Percent reduction in the extent of hypopigmented lesions (mean ± SD)	62.54 ± 17.53	93.3 ± 13.51	0.001*
Extent of hypopigmented lesions at EOS (% body surface) (mean ± SD)	3.05 ± 4.66	0.54 ± 1.49	0.086**

Percent reduction in the extent of the rash at weeks 2, 4 and 6 (mean ± SD)	Azithromycin	NB-UVB	*p* value
At week 2 (*n* = 24)			
Rash as a whole	33.33 ± 24.38	28.1 ± 13	0.512
Erythematous papules	37.15 ± 27.77	27.28 ± 19.28	0.318
Hypopigmented lesions	22.88 ± 26.6	39.9 ± 23.9	0.173
At week 4 (*n* = 23)			
Rash as a whole	55.62 ± 21.38	66.85 ± 16.72	0.163
Erythematous papules	68.7 ± 19.57	62.26 ± 17.22	0.4
Hypopigmented lesions	42.55 ± 29.22	74.56 ± 20.77	0.018*
At week 6 (*n* = 23)			
Rash as a whole	70.04 ± 19.61	84.03 ± 13	0.049*
Erythematous papules	84.26 ± 14.88	81.9 ± 16.53	0.720
Hypopigmented lesions	51.8 ± 20.46	86.8 ± 14.27	0.001*

*p value < 0.05 is significant. **Not significant but noticeable result

#### Further analysis at weeks 2, 4 and 6

**Fig. 2:**
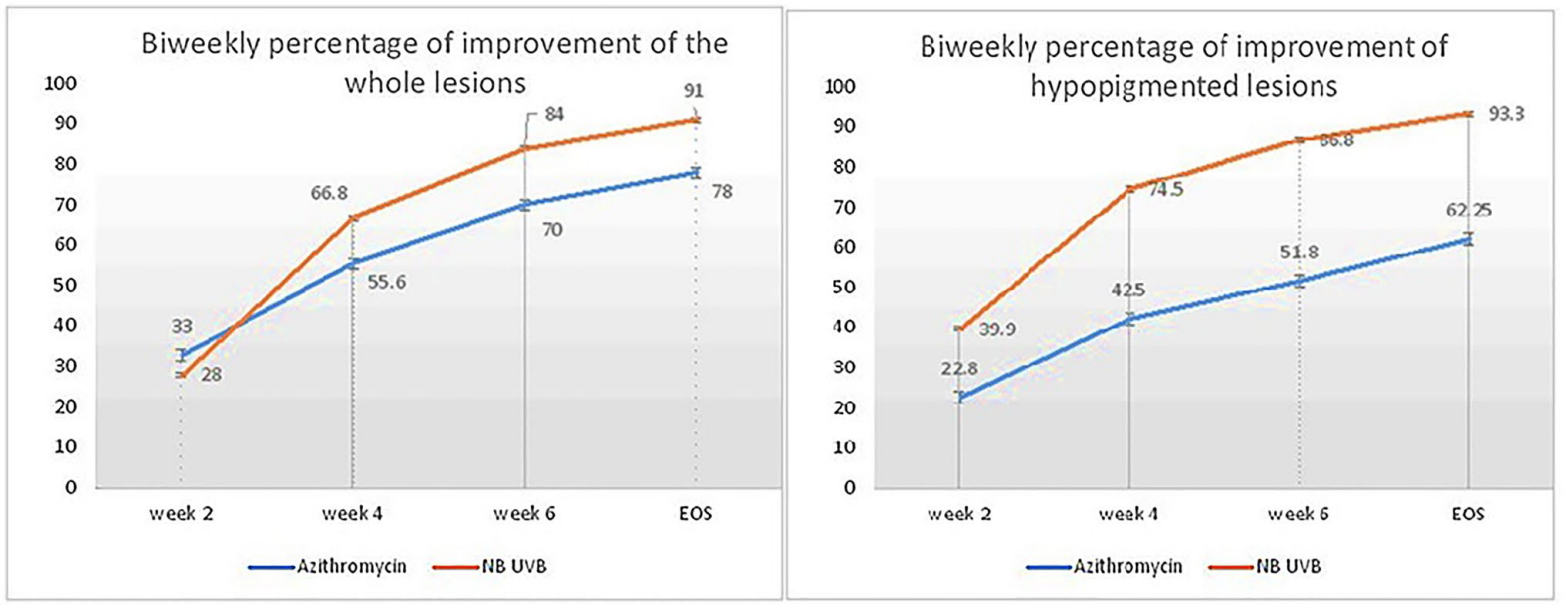
Biweekly follow-up for percent reduction in the hypopigmented lesions and the rash as a whole in azithromycin and NB-UVB groups

Comparing the percent reduction in the extent of lesions from baseline to weeks 2, 4 and 6 between both groups revealed that NB-UVB achieved significant reduction in the extent of the hypopigmented lesions in weeks 4 and 6 (*p* = 0.018 and *p* = 0.001, respectively) (Fig. 2) and in the rash as a whole in week 6 (*p* = 0.049) compared to azithromycin (Table 2).

#### Adverse events

Gastric upset was encountered in 2 patients (15.4%) in the azithromycin group. It was temporary, started 1 h after intake and lasted for few hours. It did not necessitate discontinuation of therapy. No patient developed phototoxicity or skin burn in the NB-UVB group.

### Results of follow-up after EOS

**Fig. 3:**
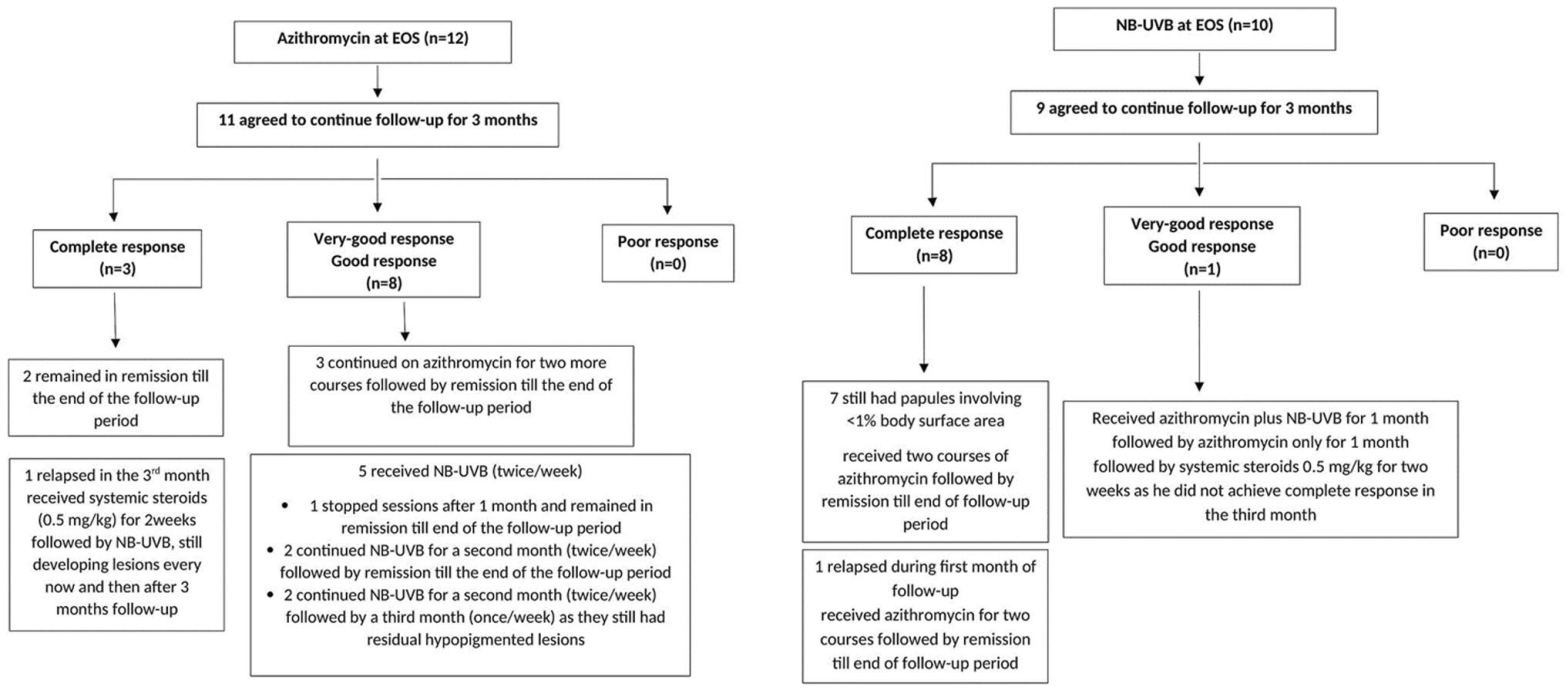
Three month follow-up for patients in the azithromycin and NB-UVB groups after EOS

Follow-up results are illustrated in Fig. 3.

### Results of streptococcal infection

A total of 19 PLC patients (79%) had evidence of streptococcal infection. ASOT was positive in 14 patients (73.7%), anti-DNase B titre was positive in 1 patient (5.3%) and both titres were positive together in 4 patients (21%). ASOT levels were > 200 IU/ml in 3(15.8%) patients, > 400 IU/ml in 14(73.7%) and > 800 IU/ml in 1(5.3%) patient. No patient had positive throat culture of group A β-hemolytic streptococci. It was significantly more encountered in young children (< 13 years) (*p* = 0.03) and was significantly associated with more extent of erythematous papules and consequently more extent of the rash as a whole at baseline (*p* = 0.05 and *p* = 0.01, respectively) (Table 3).

**Table 3:** Effect of recent streptococcal infection on the extent of the rash at baseline

	Positive recent streptococcal infection (*n* = 19)	Negative recent streptococcal infection (*n* = 5)	*p *value
Extent of the rash a whole at baseline (% body surface area)	17.64 ± 11.6	8.45 ± 4.33	0.01*
Extent of the erythematous papules at baseline (% body surface area)	10.41 ± 11.31	4.45 ± 2.40	0.05*
Extent of hypopigmented lesions at baseline (% body surface area)	7.24 ± 7.62	4 ± 5.66	0.32

^*^p value < *0.05 is significant*

Within patients with recent streptococcal infection, azithromycin did not achieve better results compared to NB-UVB (Table 4).

**Table 4:** Therapeutic efficacy of azithromycin and NB-UVB at EOS within patients with recent streptococcal infection at EOS

Therapeutic efficacy at EOS		Azithromycin	NB-UVB	*p* value
		Positive recent streptococcal infection (*n* = 12)	Positive recent streptococcal infection (*n* = 7)	
Rash as a whole	*Complete response*	2 (16.67%)	6 (85.7%)	0.106
	*Very-good response*	5 (41.67%)	1 (14.3%)	
	*Good response*	3 (25%)	0	
	*Poor response*	2 (16.67%)	0	

Therapeutic efficacy at EOS		Positive recent streptococcal infection (*n* = 12)	Positive recent streptococcal infection (*n* = 7)	*p* value
Erythematous Papules	*Complete response*	7 (58.3%)	6 (85.7%)	1
	*Very-good response*	4 (33.3%)	0	
	*Good response*	0	1 (14.3%)	
	*Poor response*	1 (8.3%)	0	

Therapeutic efficacy at EOS		Positive recent streptococcal infection (*n* = 10)	Positive recent streptococcal infection (*n* = 7)	*p* value
Hypopigmented Lesions	*Complete response*	1 (10%)	7 (100%)	0.01*
	*Very-good response*	2 (20%)	0	
	*Good response*	3 (30%)	0	
	*Poor response*	4 (40%)	0	

Percent reduction in the extent of rash and extent of rash at EOS	Positive recent streptococcal infection (*n* = 11)	Positive recent streptococcal infection (*n* = 7)	*p* value
Percent reduction in the extent of the rash as a whole (mean ± SD)	76.21 ± 13.65	93.36 ± 9.37	0.005*
Extent of the rash as a whole at EOS (% body surface) (mean ± SD)	5.5 ± 5.14	0.97 ± 1.4	0.034*
Percent reduction in the extent of erythematous papules (mean ± SD)	89.01 ± 10.38	90.12 ± 13.21	0.853
Extent of the erythematous papules at EOS (% body surface) (mean ± SD)	1.8 ± 3.57	0.85 ± 1.4	0.513
Percent reduction in the extent of hypopigmented lesions (mean ± SD)	46.9 ± 31.99	97.97 ± 3.57	0.001*

*p value < 0.05 is significant

## Discussion

Azithromycin in treatment of pityriasis lichenoides has been scarcely reported in literature and reports mainly described its efficacy in PLEVA [11, 12]. Only 2 cases of PLC treated with azithromycin were reported [13, 14]. On the other hand, phototherapy was the most studied modality in treating PLC [15], and NB-UVB was recommended as the first-line treatment in adult-onset PLC [16]. Whether NB-UVB achieves better response in treating the erythematous papular lesions or the hypopigmented lesions in PLC was not discussed in literature.

As regards therapeutic efficacy, no significant difference existed between both modalities in the current study. However, at EOS, it was observed that the percent reduction in the hypopigmented lesions (and consequently in the rash as a whole) was significantly higher in the NB-UVB compared to azithromycin (93% and 91% compared to 62.5% and 78%), an effect that has started early at week 4.

The comparable results of azithromycin and NB-UVB in the current study makes azithromycin a good therapeutic option for PLC being a more convenient modality with its daily pulsed dose that overcomes the need for regular office visits for phototherapy. However, patients presenting with hypopigmented lesions, especially when involving large body surface area, will benefit more from NB-UVB.

The follow-up data of our cohort after EOS showed that patients in the azithromycin group who still had hypopigmented lesions required NB-UVB to treat them, and some patients in the NB-UVB group benefited from azithromycin. Sequential treatment or combination of azithromycin with NB-UVB would therefor help for rapid effective treatment of PLC whenever possible.

Seventy nine percent of our cohort had evidence of streptococcal infection. The majority of cases with recent streptococcal infection were children < 13 years (73.7%). Among the positive ASOT cases, 77.7% had levels > 400 IU/ml. Although our study lacked a control population of healthy children, the percentage of cases with elevated ASOT in our cohort is quite high compared to percentages reported in healthy-looking Egyptian children [up to 400 IU/ml in 8% of children between 6 and 10 years and in 21.5% of children above 10 years and up to 800 IU/ml in 4% of children between 6 and 10 years and in 0.5% of children above 10 years] [17].

Our findings may allow PLC to be viewed as a possible post-streptococcal immune mediated disease especially that in our cohort, streptococcal infection was associated with more body surface area involvement with erythematous papules.

Streptococcus pyogenes and different strains of group A β heamolytic streptococci have been already documented to cause post-infectious non-suppurative immune mediated diseases, such as acute rheumatic fever, acute post-streptococcal glomerulonephritis, post-streptococcal reactive arthritis, guttate psoriasis, and Henoch–Schönlein purpura. These conditions appear several weeks following initial untreated or partially treated streptococcal infections [18–20].

Reports of resolution of PLC lesions after tonsillectomy, or after antibiotic intake targeting Gram positive cocci isolated from cutaneous lesions were reported [21, 22]. Despite the emerged possible role of streptococcal infection in the etiopathogenesis of PLC in our cohort, the results of subgroup analysis of patients with streptococcal infection revealed similar results to those achieved in the cohort as a whole in which favorable responses were achieved in the NB-UVB group as regards the hypopigmented lesions. This might indicate that streptococcal infection triggers cascades of immunological reactions that causes or triggers recurrences of PLC in susceptible persons and the treatment then is achieved by anti-inflammatory (immunomodulatory) agents rather than bactericidal/bacteriostatic agents hence the comparable results in both azithromycin and NB-UVB groups as azithromycin has well-known immunomodulatory effects.

Having said that, one still should raise the need for similar studies using other types of antibiotics that are more effective in treating streptococcal infection than macrolides, such as penicillin, for example.

In conclusion, therapeutic efficacy of azithromycin was found to be comparable to NB-UVB in treatment of PLC; however, NB-UVB has superior effect as regards the management of hypopigmented lesions. The high rate of associated streptococcal infection in our cohort and its correlation with the extent of the disease suggest that PLC could be a post streptococcal immune mediated disorder and warrant further studies to elaborate this association and the possible mechanisms of triggering PLC following streptococcal infection. The role of anti-streptococcal antibiotics in treating and decreasing relapses in PLC patients should be investigated.
